# Cerebellar Theta Burst Stimulation on Walking Function in Stroke Patients: A Randomized Clinical Trial

**DOI:** 10.3389/fnins.2021.688569

**Published:** 2021-10-26

**Authors:** Yun-Juan Xie, Qing-Chuan Wei, Yi Chen, Ling-Yi Liao, Bao-Jin Li, Hui-Xin Tan, Han-Hong Jiang, Qi-Fan Guo, Qiang Gao

**Affiliations:** ^1^Department of Rehabilitation Medicine, West China Hospital, Sichuan University, Chengdu, China; ^2^Department of Rehabilitation Medicine, The Third Affiliated Hospital, Sun Yat-sen University, Guangzhou, China; ^3^Key Laboratory of Rehabilitation Medicine in Sichuan Province, West China Hospital, Sichuan University, Chengdu, China; ^4^Daping Hospital, Third Military Medical University, Chongqing, China

**Keywords:** walking function, intermittent theta burst stimulation, stroke, cerebellum, neurotherapeutic

## Abstract

**Objectives:** The objective of this study was to explore the efficacy of cerebellar intermittent theta burst stimulation (iTBS) on the walking function of stroke patients.

**Methods:** Stroke patients with walking dysfunction aged 25–80 years who had suffered their first unilateral stroke were included. A total of 36 patients [mean (SD) age, 53 (7.93) years; 10 women (28%)] were enrolled in the study. All participants received the same conventional physical therapy, including transfer, balance, and ambulation training, during admission for 50 min per day during 2 weeks (10 sessions). Every session was preceded by 3 min procedure of cerebellar iTBS applyed over the contralesional cerebellum in the intervention group or by a similar sham iTBS in control group. The groups were formed randomly and the baseline characteristics showed no significant difference. The primary outcome measure was Fugl–Meyer Assessment–Lower Extremity scores. Secondary outcomes included walking performance and corticospinal excitability. Measures were performed before the intervention beginning (T0), after the first (T1) and the second (T2) weeks.

**Results:** The Fugl–Meyer Assessment for lower extremity scores slightly improved with time in both groups with no significant difference between the groups and over the time. The walking performance significantly improved with time and between group. Two-way mixed measures ANOVA showed that there was significant interaction between time and group in comfortable walking time (*F*_2,68_ = 6.5242, *P* = 0.0080, η^2^_partial_ = 0.276, ε = 0.641), between-group comparisons revealed significant differences at T1 (*P* = 0.0072) and T2 (*P* = 0.0133). The statistical analysis of maximum walking time showed that there was significant interaction between time and groups (*F*_2,68_ = 5.4354, *P* = 0.0115, η^2^_partial_ = 0.198, ε = 0.734). Compared with T0, the differences of maximum walking time between the two groups at T1 (*P* = 0.0227) and T2 (*P* = 0.0127) were statistically significant. However, both the Timed up and go test and functional ambulation category scale did not yield significant differences between groups (*P* > 0.05).

**Conclusion:** Our results revealed that applying iTBS over the contralesional cerebellum paired with physical therapy could improve walking performance in patients after stroke, implying that cerebellar iTBS intervention may be a noninvasive strategy to promote walking function in these patients. This study was registered at ChiCTR, number ChiCTR1900026450.

## Introduction

Stroke is the second most common cause of death worldwide and one of the leading causes of disability (Wang et al., 2014; Feigin et al., 2016). According to the Global Burden of Disease study of 2019, China is the country with the highest risk of stroke in the world (Langhorne et al., 2018). Even if patients are treated in time, they may still have disabilities, such as balance and walking limitations, spasms, dysphagia, and aphasia, which limit patients’ ability to carry out their daily activities and affect their quality of life (Winstein et al., 2016). Walking dysfunction is one of the most serious consequences of stroke, nearly 30% of stroke patients are unable to walk even in the chronic stage (Park et al., 2011). Therefore, recovery of walking function is strongly demanded in stroke patients.

Repetitive transcranial magnetic stimulation (rTMS) has been increasingly used to treat many neurological and neuropsychiatric disorders (Chen et al., 2019). Theta burst stimulation (TBS), a novel pattern of rTMS, saves time in the rehabilitation of motor function after stroke (Huang et al., 2005). There are two types of TBS: intermittent TBS (iTBS) and continuous TBS (cTBS) generating excitatory and inhibitory effects, respective (Larson et al., 1986; Huang et al., 2011). Compared with conventional rTMS protocols, TBS provides major advantages due to its reduced administration time (Chung et al., 2015) and long-lasting effects with lower intensity stimulation (Cárdenas-Morales et al., 2010).

Stimulation with rTMS at different sites exerts different effects depending on the impairment (Lefaucheur, 2006). The cerebellum, one of the main neural control centers for walking, plays a substantial role in movement execution and motor function, including balance, postural stability, and gait control (Bastian, 2011; Witter and De Zeeuw, 2015). Cerebellar stimulation in healthy individuals can modulate primary motor cortex excitability by altering cerebello-cerebral inhibition (Fierro et al., 2007; Langguth et al., 2008). One study demonstrated that changes in cerebellar excitability are associated with human locomotor adaptive learning, suggesting a potential role for cerebellar stimulation in stroke patients (Jayaram et al., 2011). Kim et al. (2014) reported that low frequency rTMS over the cerebellum has a curative effect on balance and walking functions in patients with ataxia following a posterior circulation stroke, further suggesting the promising therapeutic effects of cerebellar stimulation.

The research on the impact of iTBS over the cerebellum on walking performance in stroke patients is increasing. A study involving 36 patients with hemiparesis resulting from chronic ischemic strokes demonstrated that cerebellar iTBS could affect the plasticity of the cerebellar cortex and improve gait and balance function in stroke patients (Koch et al., 2018). Our previous research showed that cerebellar iTBS could improve balance function in stroke patients (Liao et al., 2021). However, the effect of cerebellar iTBS on walking function in subacute stroke patients has been rarely reported. Therefore, the purpose of this randomized, double-blind, sham-controlled study was to explore the impact of cerebellar iTBS on the walking function of stroke patients and to determine its effect on corticospinal excitability.

## Materials and Methods

### Study Design and Participants

The study was designed as a randomized, double-blind, parallel-group trial. Participants were recruited after referral to the hospital from September 2019 to September 2021. The inclusion criteria were stroke patients with walking dysfunction, which was diagnosed according to the stroke diagnostic criteria. We recruited patients aged 25–80 years (Feigin et al., 2018) who had suffered their first unilateral stroke within 6 months (Bütefisch et al., 2008), as confirmed by brain Computed Tomography (CT) or Magnetic Resonance Imaging (MRI). Exclusion criteria were having neurological disease(s) other than that the first stroke or a serious medical comorbidity (cardiac, renal or respiratory failure; active neoplasia), cerebellar or brainstem stroke, severe vision or hearing impairments, or the presence of a cardiac pacemaker, intracranial implant, or metal in the cranium. Patients with a history of seizures or who were pregnant were also excluded. The study was approved by the West China Hospital Clinical Trials and Biomedical Ethics Committee of Sichuan University. All participants were fully informed of the purpose and procedures of the study and gave written informed consent before participating in the trial.

### Randomization and Blinding

Participants were randomly assigned by a computer-generated, blockwise random sequence to either the intervention group (cerebellar iTBS coupled with physical therapy) or the control group (sham iTBS with physical therapy) with a 1:1 allocation ratio. The randomization identification number and treatment allocation code were kept in sealed opaque envelopes. Assessments were performed by two study assessors (Y-JX and L-YL) who were not otherwise involved in the study. Both assessors were trained how to administer and score the outcome measures. Participants, physical therapists, and study assessor were unaware of the group assignment. Physiotherapists who performed the cerebellar iTBS and sham iTBS were aware of the treatment condition. Participants were instructed not to discuss their treatment allocation with the treatment technicians or other participants.

### Transcranial Magnetic Stimulation Procedure

During the examination, the participants were seated in a chair and were asked to relax their arms in a comfortable position. A bathing cap with brain regions was placed on each participant’s head in order to conveniently mark the primary motor cortex. Surface electromyography was recorded from the contralateral abductor pollicis brevis (APB) muscle, using Ag-Cl electrodes and a muscle belly tendon configuration (Berger et al., 2011). The active electrode was placed over the APB muscle belly, and the reference electrode was placed on the arm, 10 cm from the wrist.

Abductor pollicis brevis muscle motor-evoked potentials (MEPs) were evoked by TMS delivered using a CCY-I magnetic stimulator (YIRUIDE medical, Wuhan, China) with a 70 mm diameter figure-of-eight coil over the contralateral primary motor cortex (M1). The intensity was initially set at 100% of the machine output to determine the optimal stimulation site (hotspot). The initial TMS coil was placed over M1 with the handle directed backward and laterally and at an angle of approximately 45° to the mid-sagittal line of the head. We determined the hot spot by moving the coil over the scalp to find the location where TMS produced the largest MEP from the target muscle during muscle activation. The hot spot was then marked on the scalp. Subsequently, we decreased the intensity in a stepwise manner while stimulating the hotspot. The resting motor threshold (RMT), which was defined as the lowest stimulus intensity to produce MEPs of at least 50 μV in at least 5 of the 10 consecutive trials, of the contralateral abductor pollicis brevis muscle was measured over the M1 of the unaffected hemisphere (Rossini et al., 2015). The active motor threshold (AMT) was defined as the lowest intensity required to evoke MEPs of greater than 200 μV in at least five out of ten trials while the subject performed a 10% of maximum voluntary contraction using visual feedback from a dynamometer (Terao et al., 1998). The AMT was only assessed once before the cerebellar stimulation to determinate the stimulation intensity of each patient.

### Interventions

Cerebellar iTBS was performed using a CCY-I magnetic stimulator (YIRUIDE medical, Wuhan, China) with a standard 70 mm diameter figure-of-eight flat coil. The stimulus intensity was set at 80% of the AMT. Each session of iTBS consisted of bursts of three pulses at 50 Hz applied at a rate of 5 Hz, with 20 trains of 10 bursts delivered at 8-s intervals, achieving 600 pulses in total. iTBS was applied over the contralesional cerebellum, 1 cm inferior to and 3 cm lateral to the inion (Del Olmo et al., 2007). Cerebellar iTBS was performed daily for 10 consecutive weekdays. The coil was positioned tangentially to the scalp, with the handle pointing upward (Pinto and Chen, 2001). Sham iTBS was delivered with the coil applied perpendicular to the scalp (Shin et al., 2019). The parameters, including noise, time, and frequency, of the sham iTBS were the same as those of the real iTBS to minimize current flow into the skull (Machado et al., 2008). After receiving cerebellar iTBS, all participants received conventional physical therapy, including motor function, transfer, balance, and ambulation training, during admission for 50 min per day. Interventions were initiated on the weekday following the pretest and were performed daily for 10 consecutive weekdays. iTBS and conventional physical therapy were conducted and supervised by well-trained and qualified physical therapist.

### Outcomes

The primary outcome measure was the Fugl–Meyer Assessment–Lower Extremity (FMA-LE), which was reported to have good reliability for evaluating lower extremity motor control in stroke patients (Sanford et al., 1993). It was scored on a 3-point ordinal scale (0–2), with a maximum score of 34. Higher scores indicated better control of the lower extremities. Secondary outcome measures included walking performance and corticospinal excitability. The assessment was performed at treatment sites before the intervention (T0), after 1 week of the intervention (T1) and after 2 weeks of the intervention (T2) by physical therapist who was unaware of the intervention assignment. Any adverse effects or discomfort reported during the iTBS sessions were investigated and recorded. The baseline assessment of stroke severity was conducted using the National Institutes of Health Stroke Scale (NIHSS) (Gandhi and Sharma, 2020).

#### Walking Performance

The ten-meter walking test (10 MWT) is a valid and reliable measure of walking ability in stroke patients (van Bloemendaal et al., 2012) that assesses the time it takes for subjects to walk 10 m at a self-selected speed and maximum speed with or without a gait aid. The Timed Up and Go test (TUG) evaluates dynamic balance and mobility function, and is reported to have excellent test-retest reliability and to correlate well with other measures of gait and balance in stroke patients (Lin et al., 2004; Flansbjer et al., 2005). TUG assesses the time taken to complete a series of actions, including standing up from a chair, walking forward three meters, turning, and walking back to the chair. The functional ambulation category scale (FAC) is a quick and cost-effective visual measurement of walking (Wade, 1992), that correlates the walking speed with the step length. The FAC has been proven to possess excellent reliability, predictive validity, and good responsiveness in stroke patients (Mehrholz et al., 2007).

#### Corticospinal Excitability

The peak-peak amplitude of MEPs were recorded by delivering a pulse at an intensity of 120% of the RMT through a figure-of-eight coil placed on the contralateral motor cortex. The average RMT, MEP amplitude were used to measure corticospinal excitability (Boylan and Sackeim, 2000). MEP measurement is a sensitive approach for detecting residual corticospinal function and is predictive of motor recovery after stroke (Peinemann et al., 2004).

### Statistical Analyses

The sample size calculation was based on data from Koch et al. (2018) showing an estimated effect size of 0.28 on the Fugl–Meyer Assessment score when comparing cerebellar iTBS with sham stimulation. To detect a significant increase from the baseline in the primary outcome measure after the 2-week iTBS intervention, it was estimated that at least 15 patients per group were needed to ensure a statistical power of 0.90 and a two-sided α significance level of 0.05. The dropout rate was expected to be 20%, on the basis of clinical experience during the study design period, so 18 patients were enrolled in each group.

The intention-to-treat population, which included all randomized patients who received at least 1 day of therapy, was used to analyze the primary and secondary outcomes. Missing outcomes data were imputed using the last observation carried forward approach. The means [standard deviation (SD)] or medians [interquartile range (IQR)] of the outcome measures are reported as appropriate. For continuous measures, the normality of the data was tested using the D’Agostino-Pearson normality test. Parametric methods were used for normally distributed data. For nonparametric data, the Mann–Whitney *U* test was used for between-group comparisons and the Wilcoxon signed-rank test was used for pairwise intrasubject comparisons. The primary outcome was analyzed by two-way mixed measures analysis of variance (ANOVA) with a between-individual factor group (iTBS and sham iTBS), and a within-individual factor time (T0, T1, and T2). The Greenhouse-Geisser correction was used when necessary to correct for nonsphericity. Tukey’s *post hoc* multiple comparison test was applied to explore the significant interactions within the groups, and Student’s *t*-test was used to examine differences between the groups. The secondary outcomes were both evaluated by two-way mixed measures ANOVA. Statistical significance was maintained at *p* < 0.05, and 95% confidence intervals were calculated. All statistical analyses and graph generations were performed using SPSS version 22.0 and GraphPad Prism version 7.0 (GraphPad Inc., San Diego, CA, United States). This study was registered at ChiCTR, number ChiCTR1900026450.

## Results

**FIGURE 1:**
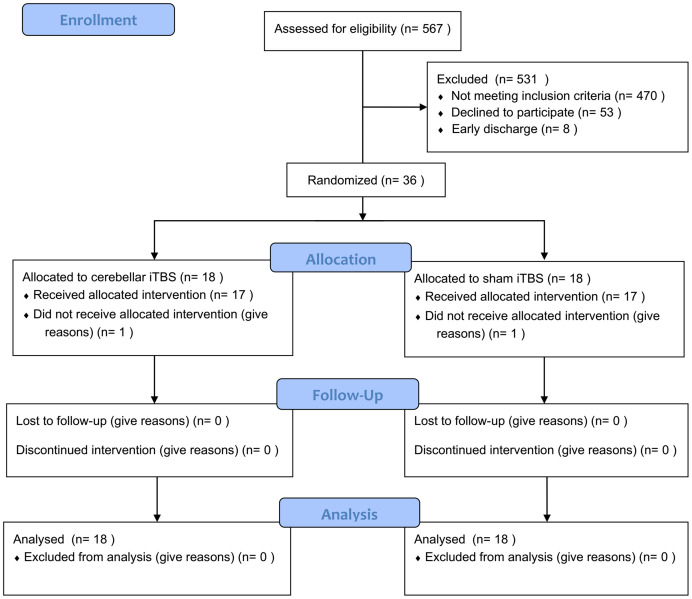
Flow diagram of patients through the study.

A total of 36 patients [mean (SD) age, 53 (7.93) years; 10 women (28%)] were enrolled in the study between September 1, 2019, and August 31, 2020 and were randomly assigned to the intervention or control group at a 1:1 ratio. However, two patients (one from each group) withdrew for personal reasons after undergoing their first evaluation but before receiving treatment. Seventeen patients in the intervention group and 17 patients in the control group completed 2 weeks of treatment and had their outcomes evaluated (Figure 1). Among those 36 patients, 20 had suffered (59%) ischemic strokes, and most participants were 1 to 6 months poststroke. There were no significant differences in age, gender, disease duration, or lesion side between the intervention and control groups. The baseline characteristics of the participants did not differ between the two groups (Table 1) and the outcome measure did not exist difference before intervention (Table 2). The mean baseline NIHSS was 4.7, with no significant difference between groups. No participants reported any adverse events.

**TABLE 1:** Baseline characteristics (*n* = 36).

**Characteristic**	**Intervention group (*n* = 18)**	**Control group (*n* = 18)**	***P* value**
Age, mean (SD), y	52.35 (8.62)	54.41 (7.01)	0.375^a^
**Sex, No. (%)**			
Male	13(72%)	11(61%)	0.480^b^
Female	5(28%)	7(39%)	
Weight, median (IQR), kg	69(64∼72)	65(58∼72)	0.437^a^
Height, median (IQR), cm	165(159∼171)	168(160∼174)	0.752^a^
**Stroke subtype, No. (%)**			
Ischemic	10(56%)	10(56%)	1.000^b^
Hemorrhagic	8(44%)	8(44%)	
**Affected side, No. (%)**			
Left	7(39%)	6(33%)	0.729^b^
Right	11(61%)	12(77%)	
Right-handed, No. (%)	18(100%)	16(89%)	0.146^b^
Time since onset, mean (SD), mo	2.22 (1.70)	2.91 (1.96)	0.233^a^
**NIHSS score, No. (%)**			
Mild (1–7)	14(78%)	15(83%)	0.674^b^
Moderate (8–16)	4(22%)	3(17%)	
Severe (> 16)	0	0	

*^*a*^Assessed using *t*-test; ^*b*^assessed using chi-square test.*

*y, year; IQR, interquartile range; mo, month; NIHSS, National Institutes of Health Stroke Scale.*

**TABLE 2:** Comparisons of outcome measures before intervention.

	**Intervention group (*n* = 18)**	**Control group (*n* = 18)**	***P* value**
FMA-LE (score)	24.94 (5.98)	23.17 (4.99)	0.339
**10 MWT(s)**			
Comfortable walking time	18.41 (9.81)	19.90 (12.53)	0.693
Maximum walking time	13.64 (6.96)	16.53 (10.95)	0.352
TUG (s)	30.25 (18.17)	36.18 (24.73)	0.418
FAC, (score), median (IQR)	3(2∼3)	3(2∼4)	0.713*
RMT (%)	45.33 (11.23)	44.00 (12.14)	0.734
MEP latency (ms)	21.12 (2.13)	21.26 (2.30)	0.857
MEP amplitude (μV)	220.89 (136.27)	197.63 (88.88)	0.548

*Data are expressed as mean (SD) unless otherwise indicated.*

*Assessed using Mann–Whitney *U*-test.

*FMA-LE, Fugl–Meyer Assessment–Lower Extremity; 10 MWT, ten-meter walking test; TUG, timed up and go test; FAC, functional ambulation category scale; IQR, interquartile range; RMT, resting motor threshold; MEP, motor evoked potential.*

### FMA-LE

**FIGURE 2:**
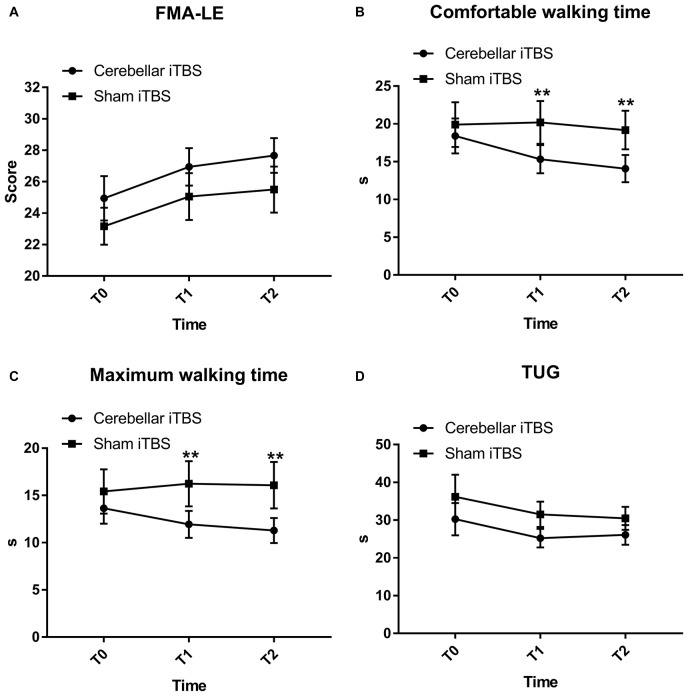
Fugl-Mayer Assessment–Lower Extremity (FMA-LE) **(A)**, comfortable **(B)** and maximum walking time **(C)** measured by ten-meter walking test, Time up and go test (TUG) **(D)** mean scores and effectiveness for the cerebellar intermittent theta burst stimulation (iTBS) and sham iTBS group at baseline (T0), 1 week after intervention (T1), and 2 weeks after intervention (T2). Error bars represent standard error of the mean (SEM). **P < 0.05.

The FMA-LE scores slightly improved with time in both groups with no significant difference between the groups and over the time [mean (SD), intervention group, T0: 24.94 (5.98); T1: 26.94 (5.05); T2: 27.67 (4.69); control group, T0: 23.17 (4.99); T1: 25.06 (6.31); T2: 25.50 (6.22)]. The analysis of the Fugl–Meyer Assessment–Lower Extremity scores showed that there was nonsphericity, and therefore the Greenhouse-Geisser correction was employed to correct the degree of freedom. The corrected results revealed a significant difference over time (*F*_2,68_ = 31.1172, *P* < 0.0001, η^2^_partial_ = 0.645, ε = 0.630), but no interaction between group and time (*F*_2,68_ = 0.1782, *P* = 0.7255, η^2^_partial_ = 0.010, ε = 0.594). The improvement trend of the experimental group was consistent with that of the control group. There was no significant difference in the main effect between the groups (*F* = 1.1440, *P* = 0.2923, η^2^_partial_ = 0.089) (Figure 2A).

### Walking Performance

#### 10 MWT-Comfortable Walking Time

The comfortable walking time decreased in the intervention group [mean (SD), T0: 18.41 (9.81); T1: 15.33 (7.92); T2: 14.08 (7.63)] but not for the control group [mean (SD), T0: 19.90 (12.53); T1: 20.20 (12.03); T2: 19.18 (10.83)]. The two-way mixed measures ANOVA showed an effect for the time factors (*F*_2,68_ = 10.2376, *P* = 0.0010, η^2^_partial_ = 0.378, ε = 0.719) and time × group (*F*_2,68_ = 6.5242, *P* = 0.0080, η^2^_partial_ = 0.276, ε = 0.641) interaction but not for the groups (*F* = 1.2851, *P* = 0.2649, η^2^_partial_ = 0.068).

The comfortable walking time decreased in the intervention group, but *post hoc* analysis revealed that the decrease within group did not reach statistical significance. The between-group comparisons revealed significant differences in the comfortable walking time at T1 (−3.37; 95% CI, −5.77 to −0.98; *P* = 0.0072) and T2 (−3.61; 95% CI, −6.42 to −0.80; *P* = 0.0133), compared to T0 (Figure 2B).

#### 10 MWT-Maximum Walking Time

The maximum walking time decreased in the intervention group [mean (SD), T0: 13.64 (6.96); T1: 11.93 (6.04); T2: 11.28 (5.63)] and the control group [mean (SD), T0: 16.53 (10.95); T1: 16.24 (10.17); T2: 16.08 (10.42)]. The corrected results using the Greenhouse-Geisser correction revealed a significant difference over time (*F*_2,68_ = 11.6524, *P* = 0.0002, η^2^_partial_ = 0.494) and interaction between group and time (*F*_2,68_ = 5.4354, *P* = 0.0115, η^2^_partial_ = 0.198, ε = 0.734) (Figure 2C).

*Post hoc* analysis showed that the mean maximum walking time differed substantially between the groups at T1 [mean (SD), 11.93 (6.04) in the intervention group and 16.24 (10.17) in the control group; mean difference, −1.41; 95% CI, −2.62 to −0.21; *P* = 0.0227] and T2 [mean (SD), 11.28 (5.63) in the intervention group and 16.08 (10.42) in the control group; mean difference, −1.91; 95% CI, −3.38 to −0.43; *P* = 0.0127] (Figure 2C) but did not differ within the groups.

#### TUG

Both patients receiving real iTBS and sham iTBS showed an improvement on the TUG [mean (SD), intervention group, T0: 30.25 (18.17); T1: 25.23 (10.37); T2: 26.09 (11.01); control group, T0: 36.18 (24.73); T1: 31.54 (14.20); T2: 30.49 (12.95), *F*_2,68_ = 5.119, *P* = 0.034, η^2^_partial_ = 0.231, ε = 0.532] (Figure 2D). However, the TUG did not display significant results time × group (*F*_2,68_ = 0.1593, *P* = 0.7163, η^2^_partial_ = 0.009, ε = 0.567) interaction or between-group differences (*F* = 1.2692, *P* = 0.2678, η^2^_partial_ = 0.078).

#### FAC

Mann–Whitney *U*-test displayed that the median FAC scores at T1 were 3 (IQR, 3 to 4) in the intervention group and 3 (IQR, 2 to 4) in the control group, but the difference between the groups was not statistically significant (0; 95% CI, −1 to 0; *P* = 0.5030). Furthermore, no significant between-group differences were found in FAC score when assessed at T2 (−1; 95% CI, −1 to 0; *P* = 0.3590). Similarly, there were no significant within-group differences.

### Corticospinal Excitability

**FIGURE 3:**
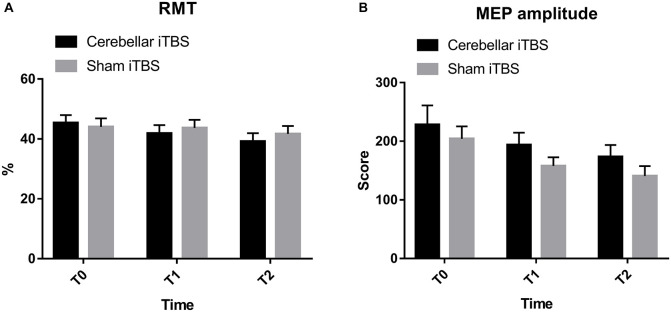
Resting motor threshold **(A)** and motor evoked potential amplitude **(B)** cerebellar intermittent theta burst stimulation (iTBS) and sham iTBS group at baseline (T0), 1 week after intervention (T1), and 2 weeks after intervention (T2). Error bars represent standard error of the mean (SEM).

The results of RMT showed that there was no significant interaction between time and group (*F*_2,68_ = 2.1638, *P* = 0.1227, η^2^_partial_ = 0.101). Over 2 weeks, the RMT in the intervention group improved from baseline in a repeated measures analysis of variance model [mean (SD), T0: 45.33 (11.23); T1: 41.83 (11.75); T2: 39.17 (11.79)], but was not statistically different compared to the control group (*F* = 0.0728, *P* = 0.7889, η^2^_partial_ = 0.007). The time difference was statistically significant (*F*_2,68_ = 9.3479, *P* = 0.0003, η^2^_partial_ = 0.387) (Figure 3A).

No increase in MEP amplitude and individual changes were found within the intervention or control group (*F*_2,68_ = 204.8659, *P* = 0.0106, η^2^_partial_ = 0.218). Besides, none of the differences between the groups were statistically significant (*F* = 1.8745, *P* = 0.1799, η^2^_partial_ = 0.129) (Figure 3B).

## Discussion

The results of this randomized, double-blind, sham-controlled clinical trial showed that in patients recovering from stroke, cerebellar intermittent theta burst stimulation plus physical therapy, compared to physical therapy alone, significantly improved walking performance, as reflected by the ten-meter walking test, comfortable walking time and maximum walking time after 1 and 2 weeks of stimulation.

These findings indicate that this 3 min cerebellar iTBS protocol, with a shorter treatment duration than the conventional rTMS protocol, improved walking function in stroke patients, a finding that is in accordance with the results of Koch et al. (2018). In our previous study, lower extremity motor function measured by FMA-LE did not improve, which was consistent with our result (Liao et al., 2021). Our research group also found that cerebellar iTBS could improve balance in subacute stroke patients (Liao et al., 2021). From a clinical perspective, cerebellar iTBS can be advantageous for designing rapid protocols for gait rehabilitation, as these improvements were achieved with a relatively short treatment duration.

Moreover, comfortable and maximum walking time decreased with cerebellar iTBS, confirming improved gait speed after intervention. Limited walking ability after stroke limits a patient’s independence in their home and community, and gait speed is the most accurate method for predicting walking classification (Perry et al., 1995). An increase in gait speed promotes a transition to improved walking, resulting in better function and quality of life, especially for those who can walk in the home (Schmid et al., 2007).

The results of the TUG revealed encouraging but nonsignificant findings suggesting better dynamic balance and mobility function following cerebellar iTBS stimulation. A study by Tramontano et al. (2020) indicated that patients receiving cerebellar iTBS showed a significant improvement in balance function. A potential explanation for the lack of positive effect on dynamic balance and mobility function in our study could be that cerebellar iTBS was applied for only 2 weeks during hospitalization.

The cerebellum is known to play a crucial role in movement execution and motor control (Manto et al., 2012). Anatomically, Purkinje cells in the cerebellar cortex inhibit the dentate nucleus, which regulates the motor cortex through the ventrolateral motor thalamus. Therefore, cerebellar brain inhibition (CBI) refers to an inhibition of the motor cortex due to activation of Purkinje cells (Ugawa et al., 1995; Daskalakis et al., 2004). It has been observed that cerebellar stimulation can modulate CBI by altering the activity of Purkinje cells, resulting in continuous and polarity-related bidirectional regulation of cerebellar excitability (Koch, 2010; Strzalkowski et al., 2019). Cerebellar iTBS could indirectly regulate the dentate nucleus by activating local low-threshold interneurons. Synapse transmission can be controlled using noninvasive brain stimulation, which results in lasting changes in synaptic connection strength. iTBS applied over the motor cortex is known to result in lasting MEP facilitation, termed long-term potentiation (LTP). The induction of LTP generates changes in activity in interconnected cortical motor networks (Koch et al., 2020).

There were no discernible differences in MEP amplitude following iTBS treatment. One possible explanation for these changes is that when the TMS-induced excitation phase is reached, that forced motor neuron excitation is more likely to result in subthreshold motor neuron discharges (Aminoff, 1986). Different intensities of 1 Hz rTMS applied over the motor cortex exert different effects (Berger et al., 2011). The MEP amplitude decreased significantly with low intensity stimulation, while high intensity stimulation increased the MEP amplitude. Additionally, an 80% motor threshold intensity resulted in less inhibition, although that decrease was not statistically significant, a finding that may also hold true in cerebellar iTBS given that no discernible differences in MEP amplitude were observed between groups.

There were several limitations to our study. First, the small sample size may affect the results of the outcome measures and did not allow for a more refined stratified analysis of the findings. Besides, only the excitability of unaffected hand M1 was assessed, which may be nonspecific for the changes. When eliminating difficulties in equipment testing, the cortical excitability of the affected hand M1, as well as the responses in the leg muscles would be more specific for the changes of corticospinal excitability. Another limitation is the lack of follow-up assessment, as we were not able to determine the long-term effects of cerebellar iTBS.

## Conclusion

Importantly, this study revealed that applying iTBS over the contralesional cerebellum paired with physical therapy could improve walking performance in stroke patients, implying that cerebellar iTBS may be a cost-effective and noninvasive strategy to promote recovery of walking function in stroke patients. More high-quality studies are needed to examine changes in corticospinal excitability.

## Data Availability Statement

The raw data supporting the conclusions of this article will be made available by the authors, without undue reservation.

## Ethics Statement

The studies involving human participants were reviewed and approved by West China Hospital Clinical Trials and Biomedical Ethics Committee of Sichuan University. The patients/participants provided their written informed consent to participate in this study.

## Author Contributions

Y-JX: conceptualization, visualization, software, validation, formal analysis, and writing—original draft. Q-CW: conceptualization, visualization, software, and validation. YC: methodology, visualization, and software. L-YL: methodology, investigation, visualization, software, writing—review and editing. B-JL and H-HJ: investigation. H-XT and Q-FG: methodology, investigation, and visualization. QG: resources, visualization, writing—review and editing, supervision, and project administration. All authors contributed to the article and approved the submitted version.

## Conflict of Interest

The authors declare that the research was conducted in the absence of any commercial or financial relationships that could be construed as a potential conflict of interest.

## Publisher’s Note

All claims expressed in this article are solely those of the authors and do not necessarily represent those of their affiliated organizations, or those of the publisher, the editors and the reviewers. Any product that may be evaluated in this article, or claim that may be made by its manufacturer, is not guaranteed or endorsed by the publisher.
